# (Non)Resonance Bonds in Molecular Dynamics Simulations: A Case Study concerning C_60_ Fullerenes

**DOI:** 10.3390/e26030214

**Published:** 2024-02-28

**Authors:** Jacek Siódmiak

**Affiliations:** Institute of Mathematics and Physics, Faculty of Chemical Technology and Engineering, Bydgoszcz University of Science and Technology, Al. Prof. S. Kaliskiego 7, 85-796 Bydgoszcz, Poland; siedem@pbs.edu.pl

**Keywords:** fullerene, resonance bonds, Shannon entropy, intrinsic vibrations, eigenfrequency, vibrational mode, molecular dynamics

## Abstract

In the case of certain chemical compounds, especially organic ones, electrons can be delocalized between different atoms within the molecule. These resulting bonds, known as resonance bonds, pose a challenge not only in theoretical descriptions of the studied system but also present difficulties in simulating such systems using molecular dynamics methods. In computer simulations of such systems, it is often common practice to use fractional bonds as an averaged value across equivalent structures, known as a resonance hybrid. This paper presents the results of the analysis of five forms of C_60_ fullerene polymorphs: one with all bonds being resonance, three with all bonds being integer (singles and doubles in different configurations), one with the majority of bonds being integer (singles and doubles), and ten bonds (within two opposite pentagons) valued at one and a half. The analysis involved the Shannon entropy value for bond length distributions and the eigenfrequency of intrinsic vibrations (first vibrational mode), reflecting the stiffness of the entire structure. The maps of the electrostatic potential distribution around the investigated structures are presented and the dipole moment was estimated. Introducing asymmetry in bond redistribution by incorporating mixed bonds (integer and partial), in contrast to variants with equivalent bonds, resulted in a significant change in the examined observables.

## 1. Introduction

Computer simulations are becoming increasingly common tools for replicating or creating reality, applicable to systems at both the atomic and macro scales [1,2,3,4,5,6,7,8,9,10]. Regardless of the method employed, they are subject to their own limitations and imperfections. In the case of molecular dynamics (MD) simulations, which have been used here, there are several issues that need careful consideration. In MD simulations, trajectories of atoms and molecules are determined by numerically solving Newton’s equations of motion for interacting particles, often calculating forces between particles and their potential energies using interatomic potentials [11,12]. The main limitation is the simulation time, which currently stands at the nanosecond scale for large systems, and we should bear in mind that this limit heavily relies on the available computational power. The restriction of nanoseconds in simulation time also imposes a constraint on the range of possible configurational sampling attainable through MD. Adequate exploration of the configuration space available under equilibrium conditions is crucial for computing thermodynamic properties linked to entropy. This is because entropy quantifies the extent of available configuration space [13].

The next issue lies in establishing an appropriately short simulation time step. Inappropriately chosen time steps lead to the accumulation of errors in numerical integration, which can be minimized with suitable algorithm selection and parameters but cannot be completely eliminated [12,14,15,16].

The next challenge is selecting an appropriate thermostat that can maintain the temperature and pressure at a constant (average) level. In MD simulations, the thermostat plays an incredibly crucial role. It is necessary for simulating isothermal-isochoric (NVT) or isothermal-isobaric (NPT) systems. To accurately simulate physical properties, the thermostat must not only sample the correct NVT/NPT ensemble but also minimally disturb particle (Newtonian) dynamics. The Berendsen thermostat is a commonly used one, as it maintains the average macroscopic temperature over time at the desired value by scaling the velocity of atoms using a weak-coupling thermostat [17]. Another frequently employed thermostat is the Lowe–Andersen thermostat, which adjusts the temperature by assigning random relative velocities to pairs of close atoms, akin to a “heat bath collision” [18].

One of the most significant, if not the most significant, challenges faced by the MD method is the appropriate selection of a force field [12,17,19,20]. The force field refers to the functional form and parameter sets used to calculate the potential energy of a system. The fundamental form of the potential energy function in molecular mechanics includes terms associated with bonded interactions among atoms connected by covalent bonds and non-bonded terms (also known as non-covalent), describing long-range electrostatic forces and van der Waals interactions. The specific distribution of terms depends on the force field, but the general form of the total energy in an additive force field can be expressed as: $E_{total}=E_{bonded}+E_{nonbonded}$. Force field functions and parameters originate from both experimental measurements and advanced quantum mechanical calculations. Full-atom force fields provide parameters to describe each atom in the analyzed system, while methods employing “united atoms” describe certain functional groups, such as methyl and methylene, in an approximate way, treating them as single atoms. On the other hand, coarse-grained force fields used in long-term simulations of proteins and nucleic acids aim for an even more simplified description of molecules, introducing pseudo-atoms to simulate the behavior of entire amino acid residues or nitrogenous bases [21]. Such approximations are necessary due to the substantial computational time required to model such large molecular systems.

Another challenge involves the presence of a solvent, typically water. There are two approaches to solvation: one can add an appropriate number of water molecules to create a fully dissolved simulation system, or alternatively, treat water as a continuous medium rather than individual molecules. A good water model must accurately reproduce six of its properties: static dielectric constant, self-diffusion coefficient, heat of vaporization, isobaric heat capacity, coefficient of thermal expansion, and isothermal compressibility. Additionally, a challenge in developing water models is to find an accurate yet simplified description of the molecule’s charge distribution that can appropriately represent hydrogen bonding in the liquid phase. Popular water models include TIP3P (Transferable Intermolecular Potential), SPC/E (Simple Point Charge), TIP4P-Ew (which improves association/dissociation balance compared to the 3-point model), OPC (Optimal Point Charges), OPC3, and OPC3-pol (Polarizable Water Model) [22,23,24,25,26].

One of the simulation methods straddling the classical and quantum descriptions is ab initio Molecular Dynamics (AIMD) simulations using the Born–Oppenheimer approximation. The Born–Oppenheimer approximation is one of the basic concepts underlying the description of the quantum states of molecules [27]. This approximation makes it possible to separate the motion of the nuclei and the motion of the electrons by neglecting the motion of the atomic nuclei when describing the electrons in a molecule. The physical basis for the Born–Oppenheimer approximation is the fact that the mass of an atomic nucleus in a molecule is much larger than the mass of an electron (more than 1000 times). Because of this difference, the nuclei move much more slowly than the electrons.

To accurately reflect real systems in all-atom MD simulations, it is necessary to consider quantum effects. Nuclear quantum effects profoundly influence the structural, thermodynamic, and kinetic properties of a wide range of chemical and biological systems. Typically, they involve zero-point effects and tunneling, which are particularly significant when light nuclei, such as hydrogen, are present. These effects are partially included during force field parameterization. The need to account for quantum effects seems crucial, especially in scenarios involving ions in a solvent, where polarization and charge transfer effects should be properly addressed [4,28].

One method used to incorporate quantum effects in MD simulations is the Adaptive Quantum Bath, which imposes a quantum energy distribution within a classical system through a generalized Langevin thermostat [29,30,31]. Another approach on the boundary between quantum and classical methods is the quantum mechanical/molecular mechanical (QM/MM) approach. In this approach, the most chemically relevant region is treated using quantum mechanics, while the remaining part is described using potential functions from molecular mechanics. Although treating subregions seems straightforward, coupling between different regions presents a challenge, especially when there is an exchange of molecules or bond crossing at the QM/MM interface.

One such phenomenon is resonance. The resonance lies at the intersection of classical and quantum understanding. Resonance is a classical concept used to describe the delocalization of electrons in molecular orbitals [32]. Resonance in chemical bonding is a concept that arises from quantum mechanics but is not strictly a quantum effect in itself. It is a way to represent molecular structures that do not correspond to a single Lewis structure, often seen in molecules where multiple equivalent structures exist. These structures represent the different ways the electrons might be distributed among the atoms in the molecule. In reality, the actual structure of the molecule is an average or hybrid of these resonance structures, and it is better explained by molecular orbital theory in quantum mechanics. Quantum mechanics helps in understanding the delocalization of electrons among different possible structures [33].

In fullerenes, carbon atoms are bonded to only three other carbon atoms. Thus, one bond must be double, but it is ambiguous which one. The number of all possible combinations where each atom forms two single bonds and one double bond is immense, growing exponentially with the size of the molecule. It is already known in the literature that there are 12,500 resonance structures of the fullerene C_60_-I_h_ [34]. Resonance bonds pose a challenge for MD simulations. One approach is to utilize fractional bonds. In MD simulation software, this is resolved by distributing the four bonds evenly among the three closest neighbors. In this study, five selected C_60_ fullerenes are analyzed. One of them exclusively features resonance bonds with a value of 1.33. In other cases, there are solely integral value bonds or mixed bonds.

The article is structured as follows: it begins with an introduction, followed by a description of the materials and methods. Subsequently, the results of the conducted simulations are presented. Finally, it concludes with a summary and conclusions.

## 2. Material

The carbon atom coordinates for fullerene C_60_-I_h_ were obtained from The Nanotube Site [35]. The fullerene geometries are based on structures in the Fullerene Library that were created by Mitsuho Yoshida and were further optimized by David Tománek using the fast Dreiding-like force field [36]. The numbering scheme of fullerene agrees with that used in the monograph “An atlas of fullerenes” by Peter W. Fowler and David E. Manolopoulos [37]. A significant characteristic of the C_60_-I_h_ molecule lies in its exceptional symmetry. Following Leonhard Euler’s theoretical evidence, a symmetrical solid (one with specific planes or axes of symmetry) containing 20 + 2*n* vertices should consist of 12 isolated pentagons and n hexagons. The smallest carbon cluster meeting this rule is C_60_ [38]. It possesses 120 symmetrical operations, such as rotation around an axis and reflection in a plane, that result in the molecule overlapping onto itself. This attribute renders C_60_-I_h_ as the most symmetric molecule within the fullerene family [33]. The fullerene C_60_-I_h_ is composed of 12 pentagons and 20 hexagons. All the rings are interconnected. Each carbon (with a valency of 4) is bonded to three others (its nearest neighbors), resulting in one of the bonds being a double bond. Because all atoms are equivalent, the double bonds are resonance bonds. Resonance is a method used to depict the distribution of delocalized electrons in specific molecules or polyatomic ions when a single Lewis structure fails to adequately represent the bonding [39]. Such molecules or ions, possessing these delocalized electrons, are depicted through multiple resonance structures. Resonance structures are used when a single Lewis structure cannot fully describe the bonding. The combination of possible resonance structures is defined as a resonance hybrid, which represents the overall delocalization of electrons within the molecule. Close to 12,500 resonance structures can be formulated for fullerene C_60_-I_h_ [40]. However, only one structure really represents it most appropriately, namely, the one in which all C=C double bonds are in the hexagons. The resonance hybrid used for studying C_60_-I_h_ has all equivalent bonds, resulting in a fractional bond order. The bond order itself is the number of electron pairs (covalent bonds) between two atoms [41]. In our case, the resonance bond order of the original configuration of C_60_-I_h_ is equal to 1.33.

**Figure 1:**
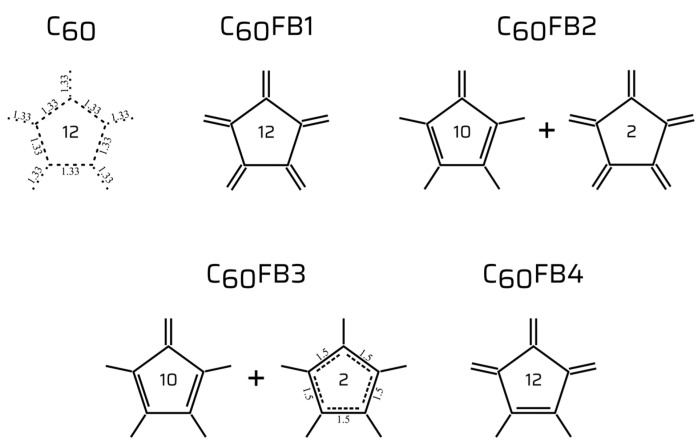
The structures of pentatomic molecules in the frame of examined C_60_ fullerenes. Solid lines represent single and double bonds, while dashed lines indicate fractional bonds (resonance). The numbers inside the pentaenes denote the quantity of pentagons (considering 20 hexagons) of a given type that form a particular fullerene structure.

In this study, five resonance forms of C_60_-I_h_ fullerene have been examined (see Figure 1). The original structure, C_60_, exhibits all bonds as resonance structures (averaged hybrid with an overall bond order of 1.33). The second, identified as C_60_FB1 (FB refers to “fixed bonds”), features all double bonds extending outward from the pentagons, and single bonds within the pentaene. The third, labeled C_60_FB2, consists of 10 pentagons with two double bonds in pentaene, one double bond outward facing, and two arranged in opposite directions pentagons, like in the FB1 form. The fourth, identified as C_60_FB3, is composed of 10 pentaenes as in FB2 and two oppositely arranged pentagons with internal resonance bonds of order 1.5. The last examined structure, labeled C_60_FB4, showcases three double bonds extending outward, including one in pentaene. The numbers inside pentaenes denote the quantity of pentagons (considering 20 hexagons) of a given type that form a particular fullerene structure. Solid lines represent single and double bonds, while dashed lines indicate fractional bonds (resonance).

The selected properties of the examined structures in their minimum energy states have been compiled in Table 1. The minimization process involves annealing and bringing the system to the nearest stable energy minimum. Radius of gyration is an imaginary distance from the axis of rotation to a point where the total mass of the body is concentrated so that the moment of inertia about the axis remains the same [42]. For a macromolecule composed of n mass elements of masses $m_{i}$, located at fixed distances $R_{i}$ from the center of mass, radius of gyration is calculated according to the formula:

(1) $$R_{g}=\sqrt{\frac{\sum_{i}^{n}m_{i}R_{i}^{2}}{\sum_{i}^{n}m_{i}}}.$$

The standard heat of formation at room temperature (298 K) $\Delta H_{f}$ is calculated using the quantum mechanics method and represents the heat released or absorbed (enthalpy change) during the formation of a molecule from its constituent elements at constant pressure. Calculations are performed by YAPAC (YASARA v22.8.22 software package, see Section 3.1), a specialized module derived from MOPAC (Molecular Orbital PACkage), originally developed by James P. Stewart, which is based on Dewar and Thiel’s NDDO approximation [43].

## 3. Methods

### 3.1. Force Field

The YASARA v22.8.22 (Yet Another Scientific Artificial Reality Application) program was used for modeling and simulation [44]. To prevent the molecule from interacting with the walls of the simulation box or itself, periodic boundary conditions were applied, with the simulation box size set to $3\times \left(100\;Å+d\right)$ (where d≈6.93 Å is the molecule’s diameter). The simulations were conducted in a vacuum using the NOVA force field, with the total energy being expressed as a sum of individual contributions (bonds, angles, planarity, Van der Waals, and electrostatic terms) [20]:

(2) $$E_{a}=\sum_{j<a}\frac{1}{2}k_{j}{\left(R_{j_{0}}-R_{j}\right)}^{2}+\frac{1}{2}l_{p}R_{p}^{2}+\sum_{i<a}\left(A_{i}e^{-B_{i}R_{i}}-\frac{C_{i}}{R_{i}^{6}}\right)+\;\sum_{b<a}\sum_{m}\sum_{n}\frac{1}{4\pi \varepsilon_{0}}\frac{q_{m}q_{n}}{R_{mn}}.$$

The energy contribution of atom a $\left(E_{a}\right)$ is the sum over all chemical bonds (to atom *j*, with bond-stretching force constant $k_{j}$, j<a), plus a planarity term $\left(0.5\cdot l_{p}R_{p}^{2}\right)$ where $l_{p}$ is a plane stretching force constant, plus the sum over all non-bonded Van der Waals interactions (with atom i, using parameters $A_{i}$, $B_{i}$ and $C_{i}$, i<a)—Born–Mayer potential with attractive $R^{-6}$ term and a short-range exponential repulsion term, plus the electrostatic Coulomb interactions between all m point charges on atom a and n point charges on atom b $\left(b<a\right)$.

### 3.2. Shannon Entropy

Shannon entropy, also known as information entropy, is used to quantify the uncertainty or average amount of information in a data set [45]. It gauges the average level of surprise, uncertainty, or information content in the data. Mathematically, it is calculated as the sum of the probabilities of each possible outcome of the random variable multiplied by the logarithm of the inverse of these probabilities. The formula for Shannon entropy of the random variable x is typically denoted as:

(3) $$H\left(x\right)=\sum p\left(x\right)\log_{2}\frac{1}{p\left(x\right)},$$

where $p\left(x\right)$ represents the probability of the *i*-th outcome and $\log_{2}\frac{1}{p\left(x\right)}$ defines the information, or surprisal, of an event x. When $p\left(x\right)$ is close to 1, the surprisal of the event is low, but if $p\left(x\right)$ is close to 0, the surprisal of the event is high. In the presented case, the dataset will consist of the lengths of all bonds between carbon atoms of a fullerene in its minimum energy state. The energy minimization procedure begins with a steepest descent minimization using the current simulation parameters. Initially, regions with strong bumps are minimized without electrostatic interactions to prevent short-range energy traps. Once the bumps are removed and the most severe conformational stress is alleviated, a simulated annealing minimization continues until the energy converges, recalculated every 200 steps.

### 3.3. Intrinsic Vibrations

To observe the intrinsic vibrations of the carbon cage, such as in fullerenes, molecular dynamics simulations were conducted using the NOVA force field, see Equation (2). The simulation lasted for 20 ns with time step equal 1 fs, allowing for the observation of vibrational modes in the range of terahertz. The control parameter was the radius of gyration $R_{g}$ of the fullerene, see Equation (1). The observed changes underwent frequency analysis using MATLAB (R2023a) [46]. The function used was fft which computes the Discrete Fourier Transform (DFT) of $R_{g}$ using a Fast Fourier Transform (FFT) algorithm [47,48]. In essence, the DFT is a mathematical operation that transforms a finite sequence of equally spaced samples of a function into its constituent frequency components. The DFT output presents a frequency-domain representation of the input signal, displaying the amplitude and phase of the diverse frequency components that form the original signal.

### 3.4. Dipole Moment and Electrostatic Potential Distribution

The electric dipole moment of the selected atoms in the local coordinate system was calculated using the charges assigned by the current force field. The electric dipole moment p is the length of the vector $\vec{P}$, which is defined as follows [49]:

(4) $$\vec{P}=\sum_{i}q_{i}\cdot {\vec{R}}_{i},$$

where $q_{i}$ is the charge of atom i, and ${\vec{R}}_{i}$ is a vector representing the position of an atom relative to the center of mass. It seems puzzling that a charge appears in a neutral molecule. This arises from the fact that in the presence of resonance bonds, not all atoms in a neutral molecule need to be neutral. The calculations are based on comparing the number of electrons in a single atom with the number of electrons in the structure. Wherever resonance bonding (partial bonding) occurs, a so-called formal charge appears [50].

A graphical representation of the electrostatic potential (ESP) inside the simulation cell based on the particle mesh Ewald approach [51]. This approach combines two techniques: the Ewald summation method and the particle mesh method. The Ewald summation method splits the electrostatic potential into two components: a real-space component and a reciprocal-space component. The real-space component is calculated by summing the contributions from the pairwise interactions between nearby particles, while the reciprocal-space component is calculated using a Fourier transform of the charge density. Compared to the real potential, this is a smoothed representation without short-range noise and singularities [52].

## 4. Results

**Figure 2:**
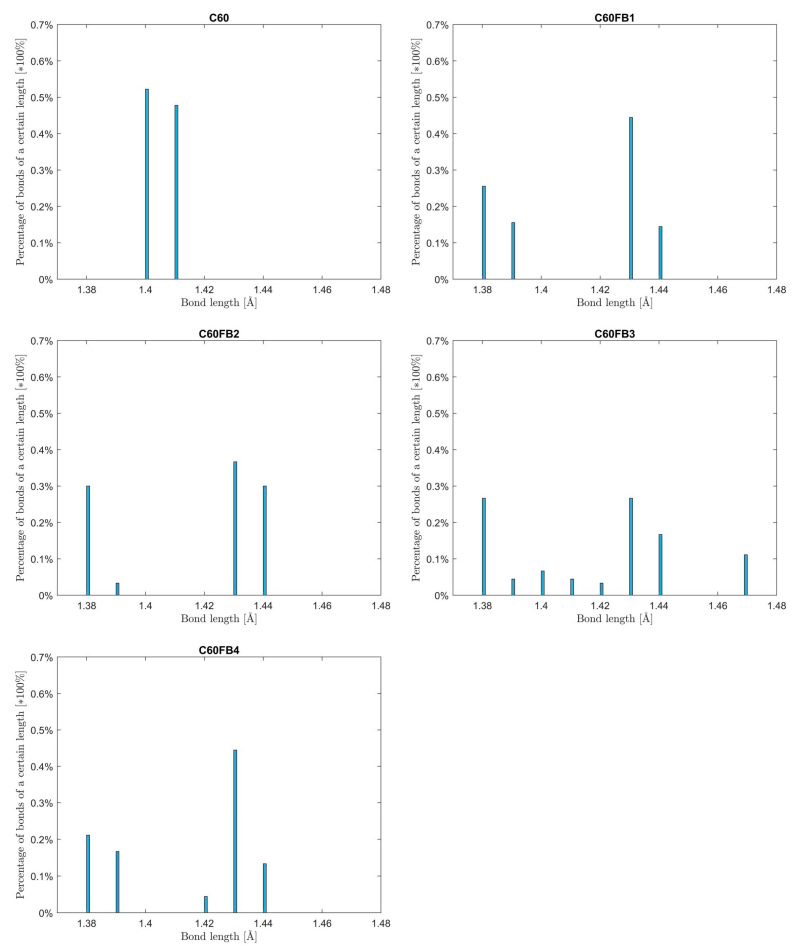
Distributions of bond lengths between carbon atoms in the examined variants of C_60_ fullerene.

Distributions of bond lengths between carbon atoms in the examined variants of C_60_ fullerene in its minimum energy state are shown in Figure 2.

As expected, in the variant of C_60_ with all equivalent resonance bonds at 1.33, all bonds are almost of the same length. The difference between them is negligible, amounting to 1/10 of an angstrom. In variants with complete single and double bonds (C_60_FB1, C_60_FB2, C_60_FB4), two groups of bonds were observed: shorter corresponding to double bonds and longer characterizing single bonds. In the case of variant C_60_FB3, which features 10 resonance bonds at 1.5, bonds of intermediate length are observed. This result aligns with observations from X-ray diffraction (XRD), nuclear magnetic resonance (NMR), neutron diffraction (ND) and gas-phase electron diffraction (EGD) experiments [53].

**Figure 3:**
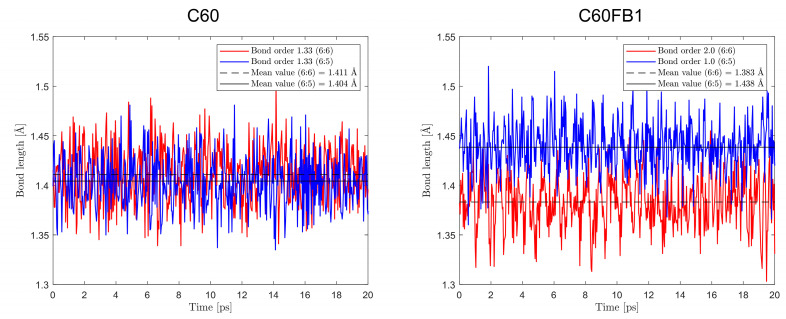
Fluctuations over time in the bond lengths of 6:6 (between hexagons) and 6:5 (between hexagons and pentagons) in the C_60_ (**left**) and C_60_FB1 (**right**) variants. Temperature 298 K.

Analysis of the bond lengths between hexagons (6:6) and between hexagons and pentagons (6:5) during an ongoing simulation at a temperature of 298 K, Figure 3, shows that in the case of C60FB1, the average bond lengths and their distribution are entirely consistent with the experimental data.

Experimental measurements conducted using various techniques indicate that the 6:6 bonds have lengths ranging from 1.39 Å to 1.40 Å, while the 6:5 bonds have lengths ranging from 1.45 Å to 1.46 Å [53,54,55,56,57].

**Figure 4:**
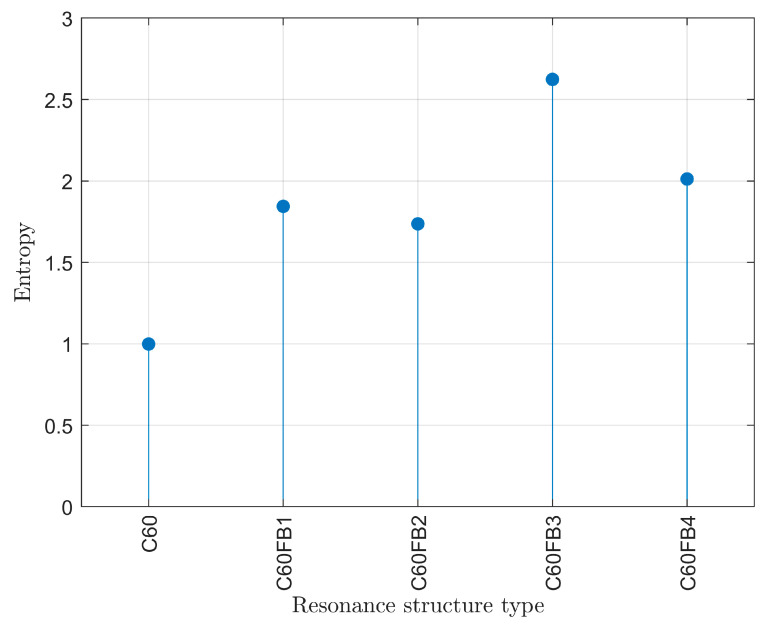
Shannon entropy for the bond lengths of the examined variants of C_60_ fullerene.

The Shannon entropy values for the bond lengths of the examined C_60_ fullerene variants are displayed in Figure 4. The structure demonstrating the highest Shannon entropy value is the C_60_FB3 variant, which exhibits the largest variety of bonds with varying lengths, although the differences in their occurrences are not substantial. This value is consistent with the histograms presented earlier.

The lowest entropy value is observed in the C_60_ variant, where all resonance bonds (valued at 1.33) are equivalent. This outcome is corroborated by the previously shown histogram. In the system where only two groups of bond lengths are nearly equally abundant, the entropy value equals 1.

**Figure 5:**
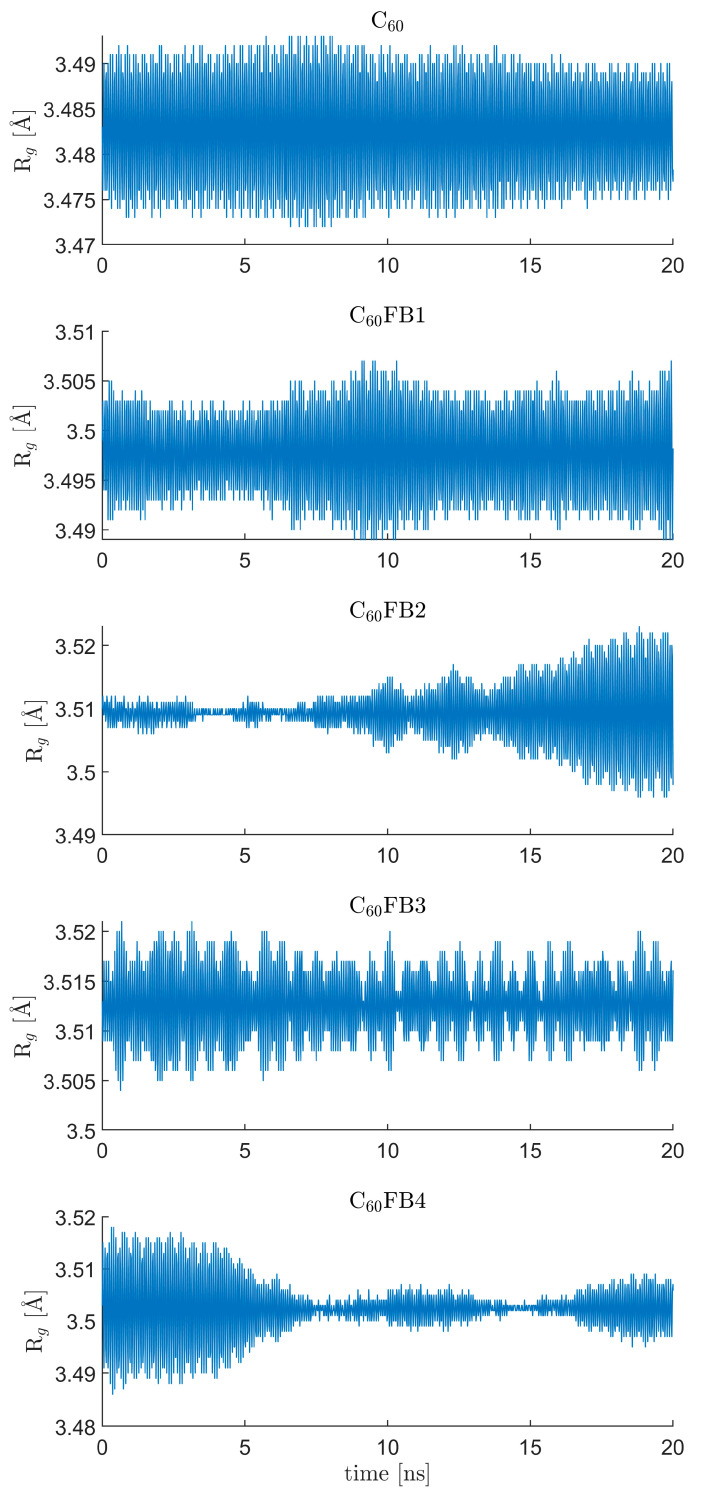
The changes over time in the radius of gyration $R_{g}$ for the examined C_60_ variants.

In Figure 5, the changes over time in the radius of gyration for the examined C_60_ variants are depicted. The simulations were conducted at a temperature of 298 K. It allows us to observe the structural fluctuations throughout the simulation. At first glance, the plotted profiles differ significantly. The overall shape of the recorded changes varied with each simulation run. What remained unchanged were the frequencies of the main vibrational modes, which are clearly visible and distinguishable.

**Figure 6:**
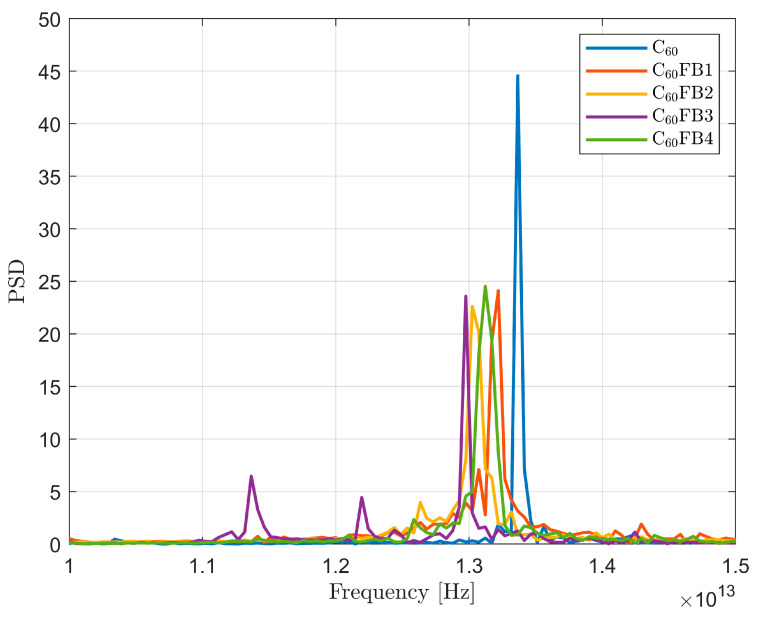
The power spectra of the time series depicting the radius of gyration changes in the examined C_60_ variants.

The FFT method was employed to analyze the DFT of the aforementioned time plots. Its outcomes are the power spectra of the analyzed signals, as presented in Figure 6. The most uniform plot, devoid of clear vibrational modes of higher order, was observed in the native variant of C_60_. In this case, one distinct peak is noticeable, significantly higher than the fundamental vibrational modes observed in the other C_60_ variants.

For the remaining structures, the power spectra are more complex. Higher-order modes are much weaker than the fundamental modes but are still discernible. An exception is the C_60_FB3 variant, where second- and third-order modes are clearly visible. This is due to the resonance bonds with a value of 1.5 located within the pentagons positioned on opposite sides of the carbon cage. Similarly, in the case of the C_60_FB2 variant, where two pentaenes that have a different distribution of double bonds than the rest of the pentagons, they are placed oppositely. However, the peak indicating the second vibrational mode is slightly weaker and closer to the main mode than in the C_60_FB3 variant.

**Figure 7:**
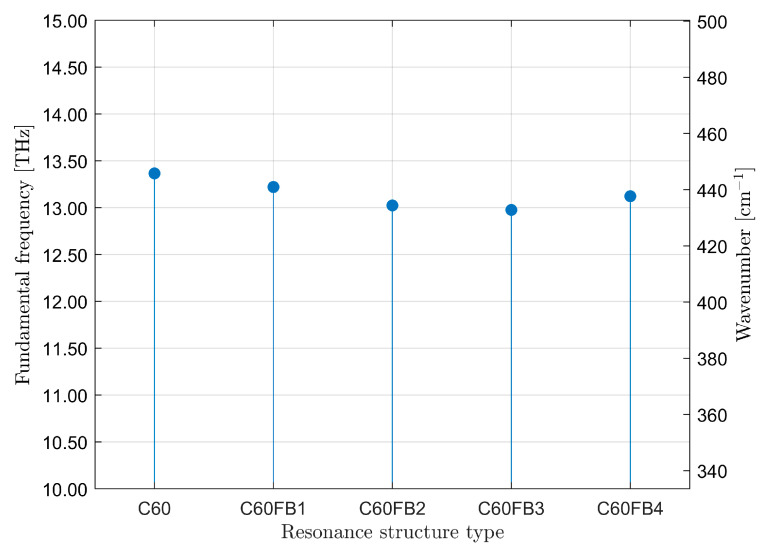
The frequencies of fundamental vibrational modes.

The frequencies of fundamental vibrational modes are summarized in Figure 7. Their values are in the range of several terahertz (about 450 cm^−1^). The difference between the highest and lowest frequencies may seem small, but it amounts to 390 GHz (about 13 cm^−1^). From the perspective of femtosecond measurement techniques, this value is significant as it allows for the unambiguous identification of the fullerene variant. It can be observed that the values of fundamental vibrational frequencies are inversely correlated with the entropy values shown in Figure 4. Lower entropy corresponds to higher frequencies of fullerene’s fundamental vibrations and vice versa. This means that greater diversity in bonds (type and length) between carbon atoms results in more complex vibrations. In turn, this means that the vibrational force is distributed among a greater number of distinct anharmonic oscillators. Similarly to bond lengths, here as well, the vibrational frequencies are approximately consistent with experimentally observed values [58,59,60,61].

**Figure 8:**
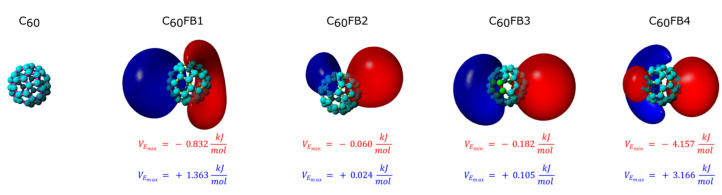
The distribution of the electrostatic potential (red - negative potential, blue - positive potential) around the examined variants of C_60_ fullerene.

The distribution of both integer and resonance bonds not only reflects in the mechanical properties, as mentioned above, but also in the electrostatic properties of the investigated molecules. It is evident that the native C_60_ variant has a zero net charge, and the charges are evenly distributed across its surface, resulting in a zero dipole moment. In the case of other variants, namely C_60_FB1-4, despite the overall zero net charge, but due to the uneven distribution of bonds, they possess a non-zero dipole moment, see Table 1, and asymmetric electrostatic potential, see Figure 8. For C60FB1-4 variants the two static surface objects are visible: first, a contour of the regions with negative potential (red); then, a contour of the regions with positive potential (blue). The contour level, which is an absolute energy value, indicates 50% of the maximum absolute electrostatic potential.

## 5. Summary and Conclusions

Computer simulations at the atomic scale, regardless of the method used, require the consideration of quantum effects. It is not a simple task, as the boundary between classical and quantum approaches is not fixed and depends on what we aim to achieve and which parameters we wish to explore. Additionally, some force field parameters are estimated based on quantum-mechanical calculations, while others rely on classical Newtonian and Coulombic interactions. Furthermore, resonance structures, which may amount to several, even tens of thousands, can significantly differ in terms of certain properties. In this study, it was shown that molecules, in this case, C_60_-I_h_ fullerenes, which differ only in the redistribution of double bonds (with the molecular formula, shape, and distribution of pentagons and hexagons forming the carbon cage remaining unchanged), exhibit varying, sometimes significant, mechanical properties. This can be observed by analyzing the power spectra of the vibrational modes and bond lengths of fullerenes. The research has shown that employing bonds with fractional bond orders in molecular dynamics simulations, as in the case of the native C60 variant, leads to bond lengths differing from the actual values observed in experiments. The disrupted symmetry in the distribution of bonds is also reflected in the electrostatic properties. Although the net electric charge remains constant and equal to zero, the mentioned asymmetry leads to an uneven distribution of this charge. Consequently, this results in the appearance of a net dipole moment and the creation of a strongly asymmetric electric field around these C_60_-I_h_ fullerene variants. These effects are not evident in the case of the native C_60_ variant, where all bonds are equivalent. Therefore, one can expect that when resonance forms differ in their distribution of electric charge, the Coulombic interactions, which act as the driving force of the aggregation process, will result in the emerging aggregates (higher-order structures) having diverse shapes and growth rates that vary depending on the direction.

## Figures and Tables

**Table 1:** Selected properties of the examined structures.

	$\mathrm{Particle}\;\mathrm{Radius}\;R_{C_{60}}$ $\left[Å\right]$	VdW Radius $R_{VdW}$ $\left[Å\right]$	Radius of Gyration $R_{g}$ $\left[Å\right]$	Potential Energy $E_{a}$ $\left[kJ/mol\right]$	$\mathrm{Heat}\;\mathrm{of}\;\mathrm{Formation}\;\Delta H_{f}$ $\left[kJ/mol\right]$	Net Charge Q $\left[C\right]$	Dipole Moment P $\left[C\cdot m\right]$
C_60_	3.47	5.33	3.44	2208	5181	0	0
C_60_FB1	3.53	5.39	3.50	3186	4477	0	5.07 × 10^−31^
C_60_FB2	3.54	5.40	3.51	3175	4644	0	1.40 × 10^−32^
C_60_FB3	3.56	5.43	3.51	2914	4866	0	7.71 × 10^−32^
C_60_FB4	3.55	5.41	3.50	3184	4527	0	1.04 × 10^−30^

## Data Availability

No new data were created or analyzed in this study. Data sharing is not applicable to this article.
